# The Impact of COVID-19 on the Treatment of Craniomaxillofacial Trauma and Head and Neck Infections

**DOI:** 10.1055/s-0040-1721426

**Published:** 2020-11-26

**Authors:** Heather A. Levites, Nathaniel L. Quinley, David B. Powers

**Affiliations:** 1 Division of Plastic, Maxillofacial & Oral Surgery, Duke University Hospital, Durham, North Carolina

**Keywords:** craniomaxillofacial trauma, odontogenic Infection, COVID, PPE

## Abstract

There is no shortage of news, information, and guidelines with regards to novel coronavirus (COVID-19). However, there is none yet that is specific to the treatment of patients who have sustained trauma or active head and neck infections—frequently encountered from oropharyngeal sources such as peritonsillar abscess or odontogenic infections. The COVID outbreak has not diminished the incidence of these conditions, and in fact has exacerbated access to care by the closing of urgent care treatment centers as well as private dental offices. The purpose of this article is to outline a protocol to protect health care providers in the provision of this care for at-risk patient populations.

There is no shortage of news, information, and guidelines with regards to novel coronavirus (COVID-19). However, there is none yet that is specific to the treatment of patients who have sustained craniomaxillofacial (CMF) trauma or active head and neck infections—frequently encountered from oropharyngeal sources such as peritonsillar abscess or odontogenic infections. The American Society of Plastic Surgeons (ASPS) released a statement on March 19, 2020 stating, “in accordance with the new Centers for Medicare and Medicaid Services (CMS) recommendation that all elective surgeries, nonessential medical, surgical, and dental procedures to be delayed during the COVID-19 outbreak, the ASPS recommends that all plastic surgeons cease providing any elective or nonessential services.” 1 The question of what is considered “essential,” however, does not have uniformity within the CMF trauma community. Otolaryngologists, plastic surgeons, and oral and maxillofacial surgeons (OMS) all provide surgical care to patients who are the victims of CMF trauma, and otolaryngologists and OMS routinely treat head and neck infections. As any surgeon who manages fractures might appreciate, these surgical interventions are arguably time-sensitive if we are to provide what is considered “standard of care” to these patients. In many cases, waiting weeks, if not months, to repair fractures of the maxilla, mandible, orbits, or midface would leave a patient incapacitated from a masticatory or visual standpoint, necessitating more involved surgical procedures in the future to correct these deformities.

Uniform guidelines for surgical repair in the CMF region are lacking. We wish to share our current practice for the protection of CMF trauma surgeons in response to the COVID-19 outbreak, in hopes that we may:

(1) Shed light on the uniquely increased risks of surgery in these regions due to the increased viral load in the oral cavity and orbital regions.

Provide a starting point for other institutions to consider when implementing their own best practices in the surgical care of CMF trauma patients.

The Australian Society of Head and Neck Surgery issued a statement on March 20, 2020 urging otolaryngologists to not only abide by the standard COVID-19 airborne precautions (good hygiene, cover cough, wash hands, avoid touching your face, self-isolate, and socially distance if ill) but also warning there is a unique risk to operating in the head and neck region and additional precautions must be taken. 2 Guidance in the United States includes the use of extreme caution when performing procedures through transnasal/transoral routes, and surgical procedures should be performed only after ascertaining the COVID-19 status and, if positive, performed only with powered airway purifying respirators (PAPR). 3 Evidence from China highlights this need; there are reports that one sinus case resulted in the infection of 14 people in the operating room and the death of multiple members of the surgical team. 4 A high rate of transmission of COVID-19 to otolaryngologists has been reported not only in China but also in Italy and Iran, many resulting in death. 5 6 7 Current advice in the United States is that preoperative COVID-19 status should be prioritized for all procedures involving the upper and lower airway and in COVID-19 positive patients, consistent with surgical plans. 3 We propose that similar precautions be taken when operating in and around the mouth and sinuses of facial trauma patients. Viral density is greatest in the nose and nasopharynx and instrumentation in and through these areas would not surprisingly lead to increased risk of transmission. The conclusion precautions similar to nasal sinus surgery should be taken when performing management of facial fractures or head and neck infections is not to be exaggerated. Osteotomizing or violating potentially infected mucosal tissue for the purposes of traumatic fracture fixation or infection management is equivalent to the powered microdebriders/shavers used in sinus surgery. All of the aforementioned surgical techniques promote possible infectious microdroplet diffusion through the operating room.

At our institution we have consistently used rapid viral/COVID testing prior to the urgent surgical care of the traumatic facial injuries, or head and neck infections, consistent with access issues to these testing platforms encountered by many health care institutions in the United States. Depending on the specific testing platform used, obtaining the results of these rapid viral/COVID tests can range from 60 minutes to 3 to 5 days. 8 Ideally, the treatment should be delayed, if the patient's physical condition or injury status allows, to perform two negative tests obtained 48 to 72 hours apart if the history is suggestive of potential exposure to account for a detectable increase in viral loads for otherwise asymptomatic patients deemed to be at high risk for COVID. Should the repeated testing confirm a negative status, or you have a negative test in a low-risk patient, performance of the surgical procedure under standard protocols of personal protective equipment (PPE) use should suffice. In patients with life-threatening head and neck infections or complex, open CMF wounds, delay may not be possible. Therefore, we propose, due to the extremely high-risk nature of oropharynx and nasopharynx manipulation, all precautions should be taken to protect the surgical providers and operating room staff involved in these cases. For patients with fever and cough/shortness of breath, high-risk travel or known COVID-19 positive status/contacts, the use of appropriate PPE is recommended for the surgical team, with use of a PAPR to protect the surgical team. As studies have shown that COVID particles with potentially infectious viral load levels have been isolated in the air after aerosolization procedures for up to 3 hours, the use of PPE is critical. 9 Patients under investigation (PUI) or emergency patients who present with immediate surgical treatment needs should be considered as COVID positive until proven otherwise. The status of any patient considering delayed or “timing elective” surgical care should be definitively identified with COVID-19 laboratory testing prior to surgical care. The ability to utilize resources for sequential negative tests prior to surgery will be limited by each facility, and a risk assessment will need to be performed by the surgical/anesthesia team for each patient should the initial testing be negative and the patient's risk profile is deemed low.

As current access to PAPRs is limited for the foreseeable future, and will be required for use on COVID-19 positive patients, this necessitates taking advantage of otherwise readily available resources to protect the CMF trauma team for patients who have either initially tested negative, or are not suspected for COVID-19. One of these is the Flyte Personal Protection System (Stryker Corporation; Kalamazoo, MI) which is generally available at health care institutions with an active orthopaedic surgery practice. At our institution, each member of the CMF trauma surgical team, including the surgical techs, are required to wear both N95 masks (3M; Saint Paul, MN) disposable shoe covers, double surgical gloves, and the Flyte helmet for additional protection against potential aerosolized tissue with high viral loads ( Fig. 1 ). Due to the difficulty in obtaining adequate quantities of the N95 masks, Duke University had previously published criteria for the reuse of these masks without degradation in the protective properties. 10 The Flyte system provides American Association for Advancement of Medical Instrumentation Level-IV protection against blood-borne and viral pathogens. While not as secure as a dedicated PAPR, the Flyte system provides superior protection by the inclusion of a clear face shield and cowl which prevents exposure to the orbits, oral/nasal cavities, and skin of the face and neck. Although the Flyte system and similar devices are not specifically designed for the protection of airborne pathogens, this system is readily available and likely affords additional protection in lower risk patients. Limitations to the Flyte system include the inability to wear a surgical headlamp on some models without a built-in light ( Fig. 2 ), and the inclusion of a motorized fan which circulates air from the operating room within the helmet. Although the fan is directed at the back of the head away from the operative field, which should limit inclusion of aerosolized particles. As such, Duke University has recently published modifications to the Flyte system which serve to decrease the potential for inhalation of aerosolized contagions to levels consistent with high-efficiency particulate air filtration. 11

**Fig. 1:**
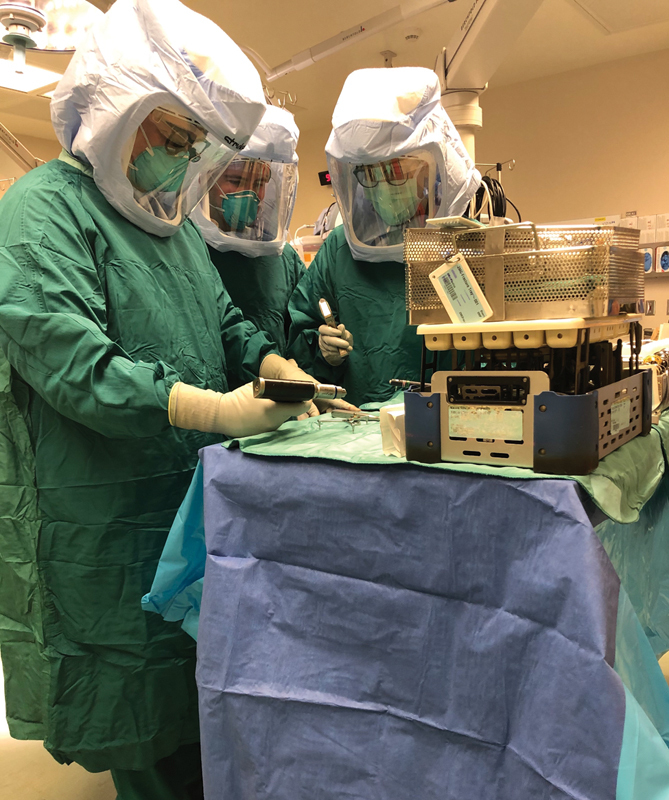
Attending surgeon (left) along with physician assistant (center) and surgical technician (right) wear an N95 respirator along with a Flyte hood (Stryker Corporation; Kalamazoo, MI) for protection against aerosolized particles during the open reduction and internal fixation of a displaced zygomaticomaxillary complex fracture. Of note the surgical technician is wearing a conventional surgical mask over her N95 mask (3M; Saint Paul, MN) as barrier protection to prevent soiling of the N95 mask, which is currently in limited supply.

**Fig. 2:**
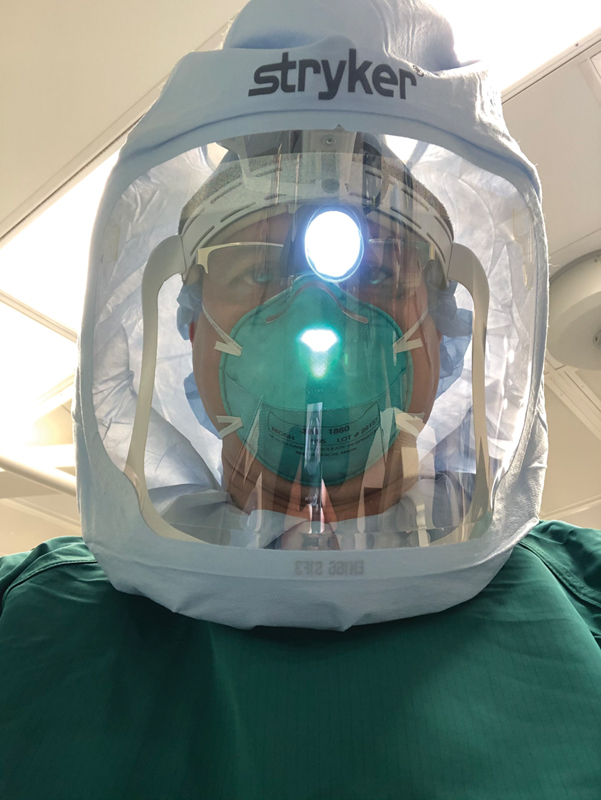
Flyte hood with attached headlight.

Our specific recommendations for the use of PPE in treatment of CMF trauma or head and neck infections at this time are discussed below.

## Recommendations Concerning CMF Trauma Procedures (Adapted from the AO Foundation Best Practices Recommendations during COVID-19 Pandemic) 12

### Lower Facial/Mandible Fractures

1. Consider closed reduction with self-drilling maxillomandibular fixation (MMF) screws.
2. Scalpel over monopolar cautery for mucosal incisions.
3. Bipolar cautery for hemostasis on lowest power setting.
4. Self-drilling screws for monocortical screw fixation.
5. When drilling is required, limit or eliminate irrigation.
6. If drilling is required, consider a battery-powered low-speed drill.
7. If a fracture requires open reduction and internal fixation, consider placement of MMF screws intraorally, then place a bioocclusive dressing over the mouth, and use a transcutaneous approach rather than an extended intraoral approach.
8. If osteotomy is required, consider osteotome instead of power saw.

### Midface Fractures

1. Consider closed reduction alone if fracture is stable following reduction.
2. Consider using Carroll–Girard screw for reduction, and avoid intraoral incision, if two-point fixation is sufficient for stabilization.
3. Scalpel over monopolar cautery for mucosal incisions.
4. Avoid repeated suctioning/irrigation.
5. Bipolar cautery for hemostasis on lowest power setting.
6. Self-drilling screws preferred.
7. If osteotomy is required, consider osteotome instead of power saw or high-speed drill.

### Upper Face Fractures/Frontal Sinus Procedures

1. Consider delay of nonfunctional frontal bone/sinus fractures.
2. Endoscopic endonasal procedure, and the associated instrumentation (power microdebriders) carry a high risk of aerosol generation and should be avoided if possible.
3. When performing a frontal sinus obliteration or cranialization consider performing the mucosal stripping manually, and not using a bur or power equipment.
4. Avoid repeated suctioning/irrigation.
5. Bipolar cautery for hemostasis on lowest power setting.
6. Self-drilling screws preferred.
7. If osteotomy is required, consider osteotome instead of power saw.

## Recommendations Concerning Oral Surgical Procedures (Adapted from American Association of Oral and Maxillofacial Surgeons March 17, 2020) 12

1. Emergency and urgent care should be provided in an environment appropriate to the patient's condition, and with appropriate PPE. Recall that any procedure involving the oral cavity is considered high risk.
2. Asymptomatic patients requesting removal of disease-free teeth with no risk of impairment of the patient's condition or pending treatment should defer treatment to a later date.
3. Asymptomatic patients, PUI, and patients tested positive for COVID-19, who have acute oral and maxillofacial infections, active oral and maxillofacial disease, should be treated in facilities where all appropriate PPE, including N95 masks, are available.
4. Patients with conditions in which a delay in surgical treatment could result in impairment of their condition or impairment of pending treatment should be treated in a manner as quickly as possible with appropriate PPE.

At this time, delaying operative fixation of unstable facial fractures or head and neck infections is not recommended; however, each case should assessed by a health system-specific team for suitability for surgical treatment.
